# Supplementary material for: Real-time dynamics and structures of supported subnanometer catalysts via multiscale simulations
Source: Nat Commun. 2021 Sep 14;12:5430. doi: 10.1038/s41467-021-25752-8 (PMC8440615; doi:10.1038/s41467-021-25752-8)
Supplement: Supplementary file 1 — Supplementary Information [file 41467_2021_25752_MOESM1_ESM.pdf]

Supplementary Information for

Real-time Dynamics and Structures of Supported Subnanometer Catalysts via Multiscale Simulations

Yifan Wang,[1,2†] Jake Kalscheur,[1,2†] Ya-Qiong Su,[3,4†] Emiel J.M. Hensen,[4*] and Dionisios G. Vlachos[1,2*]

[1]Department of Chemical and Biomolecular Engineering, 150 Academy St., University of Delaware, Newark, Delaware 19716, United States

[2]Catalysis Center for Energy Innovation, RAPID Manufacturing Institute, and Delaware Energy Institute (DEI), 221 Academy St., University of Delaware, Newark, Delaware 19716, United States

[3]School of Chemistry, Xi'an Key Laboratory of Sustainable Energy Materials Chemistry, State Key Laboratory of Electrical Insulation and Power Equipment, Xi'an Jiaotong University, Xi'an 710049, China

[4]Laboratory of Inorganic Materials and Catalysis, Department of Chemical Engineering and Chemistry, Eindhoven University of Technology, P.O. Box 513, 5600 MB Eindhoven, The Netherlands

*Corresponding authors: e.j.m.hensen@tue.nl, vlachos@udel.edu

†These authors contributed equally: Yifan Wang, Jake Kalscheur, Ya-Qiong Su

Supplementary Figures

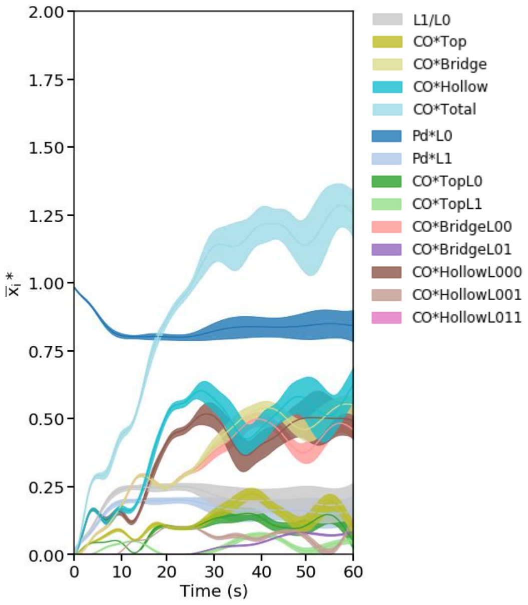

Supplementary Figure 1. Species ratios as a function of time (in the first 60 s) starting with 20 single atoms at 300 K, CO partial pressure of 0.1 bar. The means and 95% confidence intervals over triplicate runs are shown as solid lines and shaded areas, respectively.

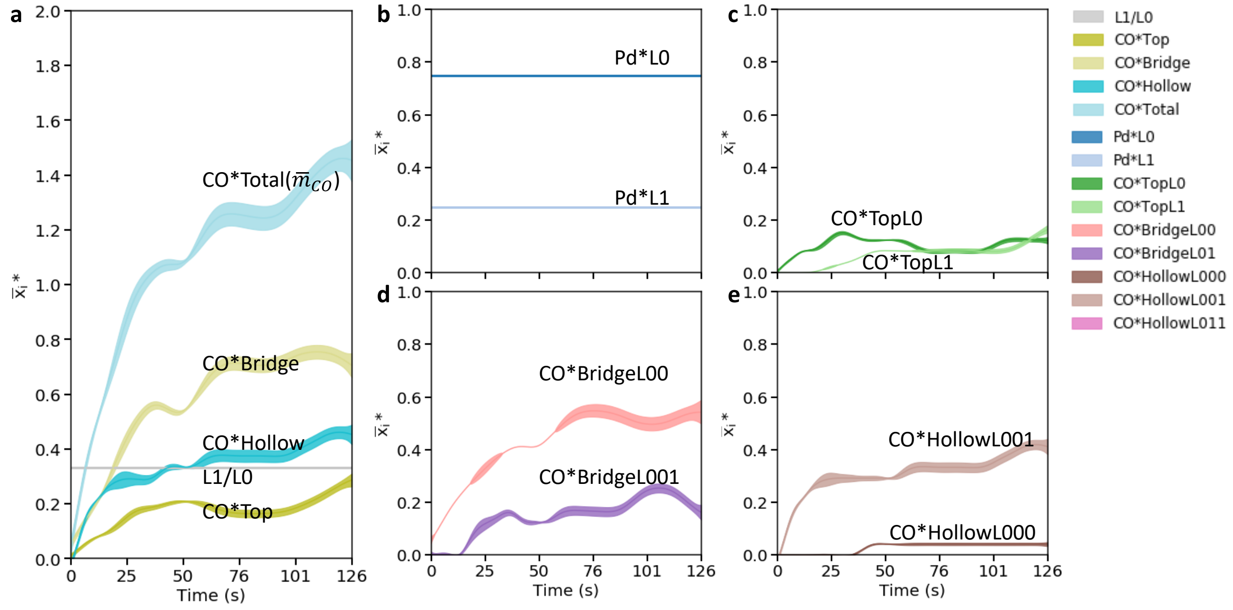

Supplementary Figure 2. Species ratios as a function of time starting with 6 Pd_4_3d clusters at 300 K, CO partial pressure of 0.1 bar.

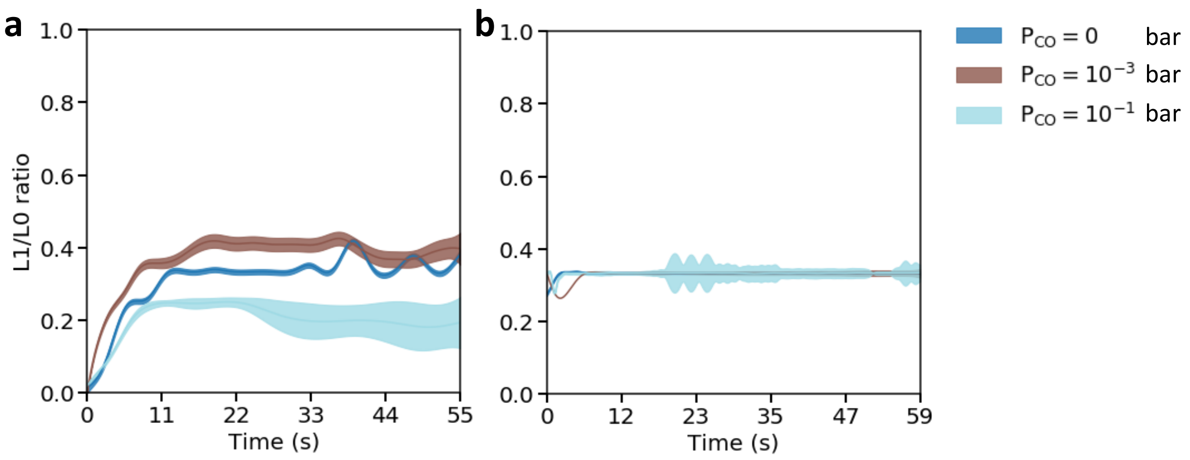

Supplementary Figure 3. L1/L0 ratios for the systems starting with (a) 20 single atoms (b) 6 Pd_4_3d clusters.

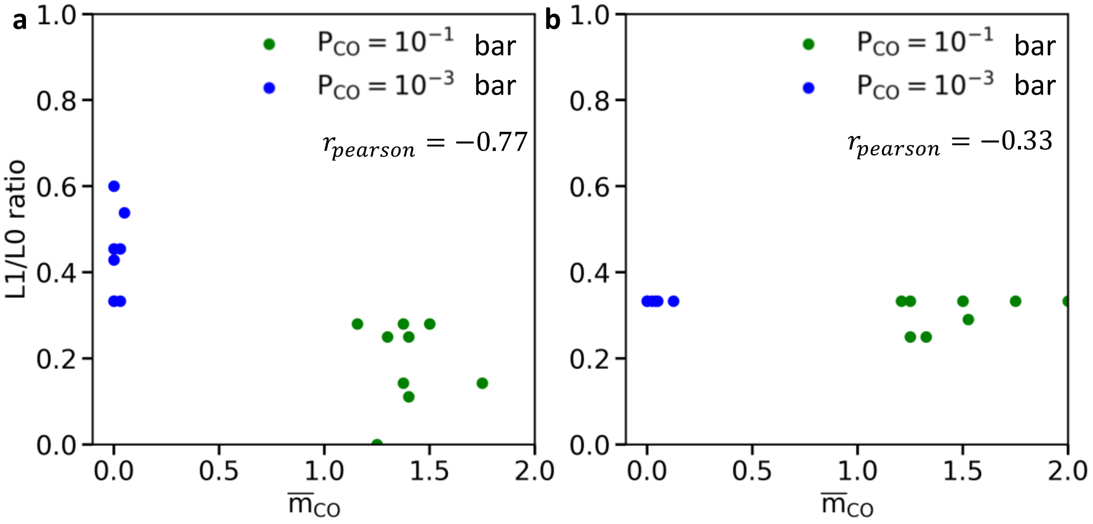

Supplementary Figure 4. Final CO loadings ($\overline{m}_{CO}$) vs. L1/L0 ratio for systems starting with (a) single-atoms and (b) Pd_4_3d clusters.

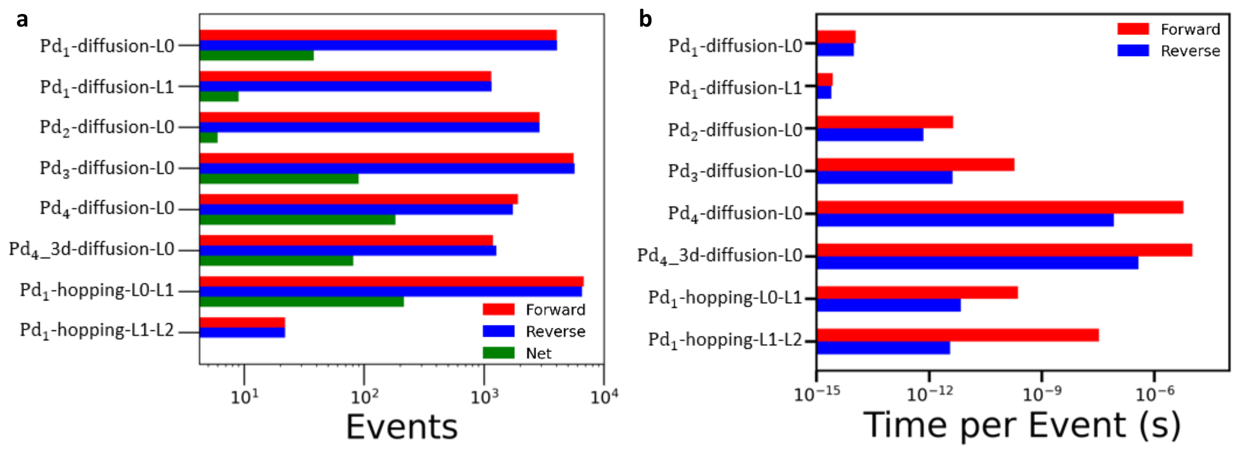

Supplementary Figure 5. (a) Event frequency and (b) average time per event in the simulation with an initial state of 8 single atoms at 700 K, CO partial pressure of 0.1 bar at long times.

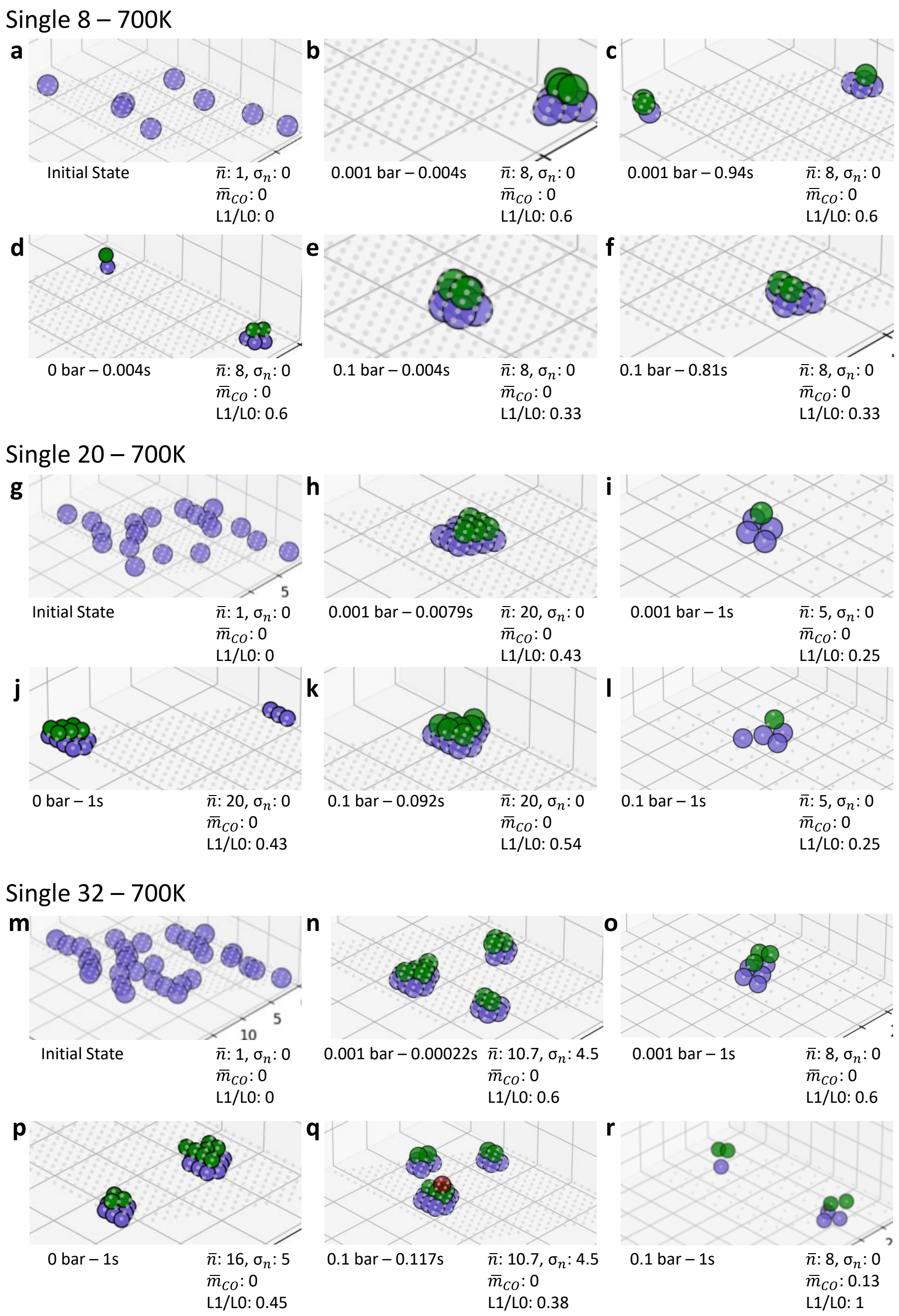

Supplementary Figure 6. Snapshots with an initial state of (a-f) 8, (g-l) 20, (m-r) 32 single atoms at 700 K, and various CO partial pressures. The third column shows the snapshots from the simulations with the longest KMC time achieved. Due to fast diffusion events, simulations at higher temperatures progress very slowly in terms of the time clock. To reach 1 s, we reduce the lattice size to 10 by 10 and the number of Pd atoms by a factor of 4 in (i), (l), (o) and (r) while keeping the same coverages. Color code: gray, empty lattice sites; red, CO; purple, Pd L0, green: Pd L1. Notations: $\bar{n}$ -average cluster size, σ_n - standard deviation of cluster size, $\bar{m}_{CO}$ – the CO loading, L1/L0 ratio – the ratio between the number of Pd atoms in L1 and the number in L0 sites.

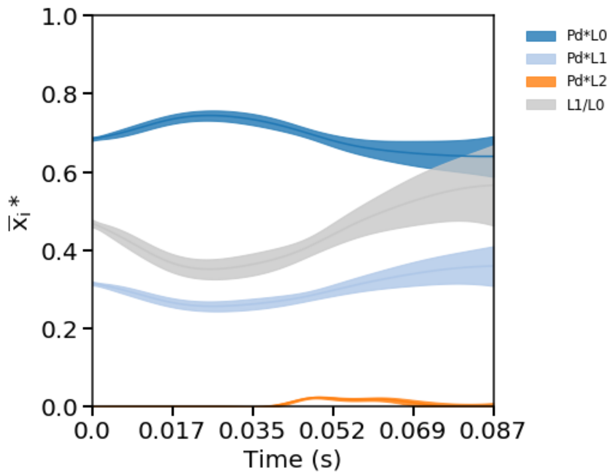

Supplementary Figure 7. Species ratios as a function of time starting with 20 single atoms at 700 K, CO partial pressure of 0.1 bar.

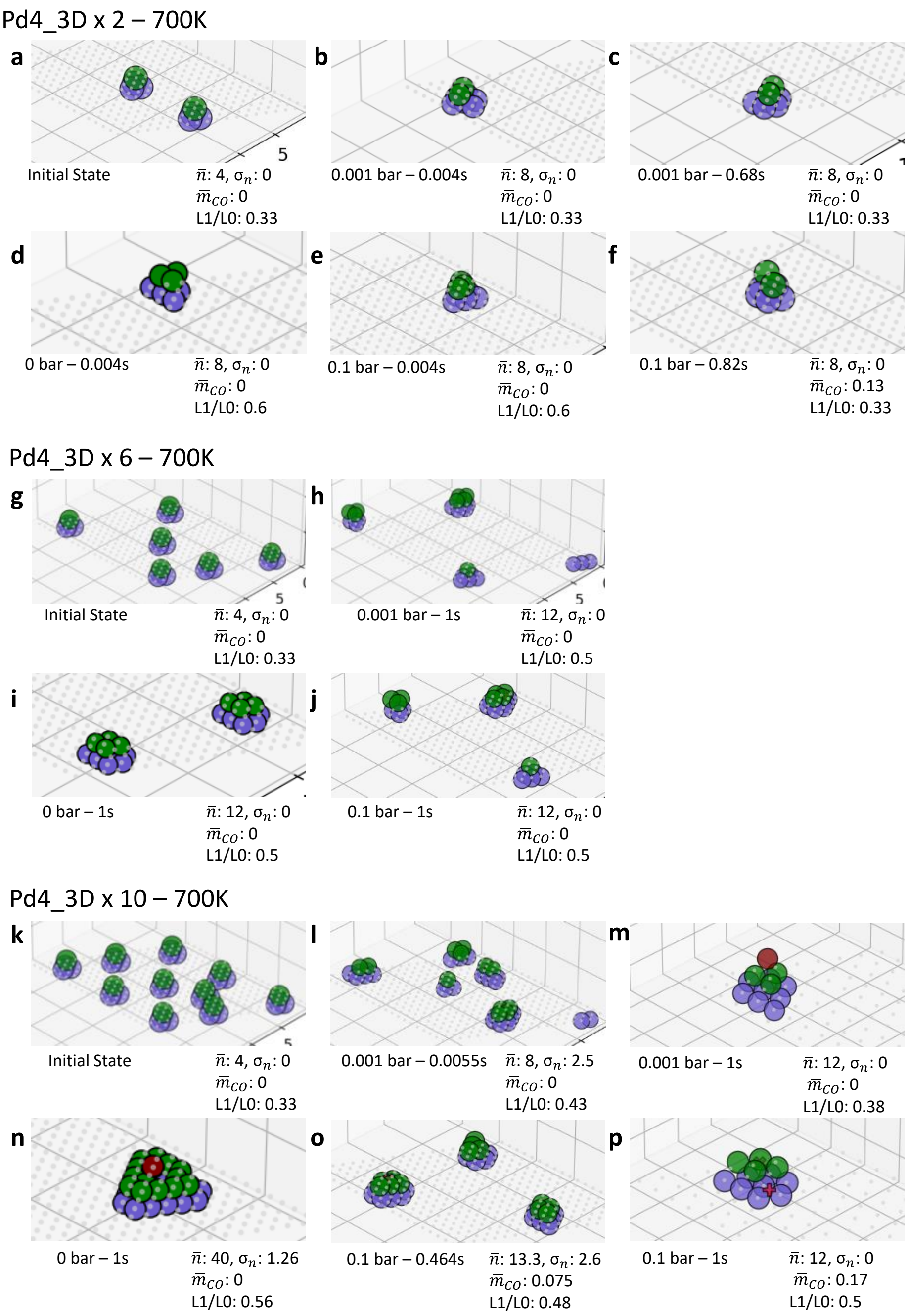

Supplementary Figure 8. Snapshots from an initial state of (a-f) 2, (g-j) 6, (k-p) 10 Pd$_4$_3d clusters at 700 K, and varying CO partial pressure. To reach 1 s, we reduce the lattice size to 10 by 10 and the number of Pd atoms by 4 in (c), (f), (m) and (p) while keeping the same coverages.

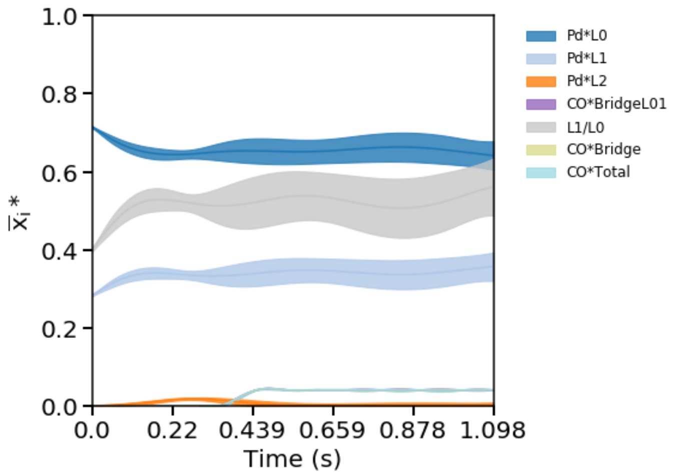

Supplementary Figure 9. Species ratios as a function of time starting with 6 Pd_4_3d clusters at 700 K, CO partial pressure of 0.1 bar.

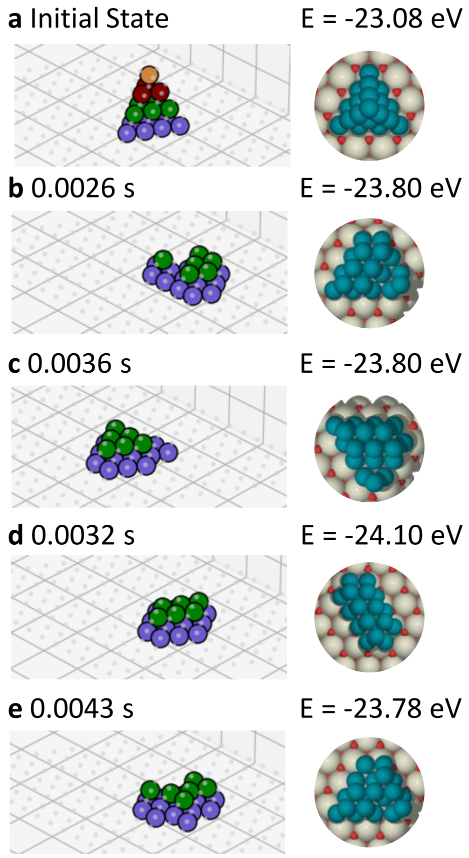

Supplementary Figure 10. Snapshots from KMC (left column) with an initial state of a Pd_{20}- pyramid structure at 300 K, CO partial pressure of 0.1 bar. Corresponding metastable structures (right column) from structure optimization based on thermodynamic stability. The cluster energies relative to 20 Pd single atoms are shown. (d) is the stable (global optimal) structure for n=20.

Supplementary Note 1 - DFT Calculations

We carry out spin-polarized calculations within the density functional theory (DFT) framework as implemented in the Vienna *ab Initio* simulation package (VASP). The projector-augmented wave (PAW)[1] method and the Perdew-Burke-Ernzerhof (PBE) exchange-correlation functional[2] are adopted. The DFT+U approach is used to model Ce. The long-range van der Waals interactions are included by setting the IVDW in the INCAR for all the DFT calculations to 12 (the DFT-D3 method with the Becke-Jonson damping is employed). We perform vibrational model analysis to verify the identified transition states. The detailed parameters and setup are as in our prior work.[3]

Supplementary Note 2 - Training of Hamiltonians *via* Machine Learning

We develop two machine learning Hamiltonian models trained on DFT data for Pd cluster energy H_{Pd_{n-i}/CeO_2} and CO adlayer energy $H^{CO-ads}_{Pd_{n-i}/CeO_2}$, respectively, where Pd_{n-i} stands for a structure (the ith isomer) of the cluster of n Pd atoms. Each Hamiltonian model is built using the cluster expansion (CE) approach, where the energy is written in terms of contributions from cluster patterns of various sizes (single, two-body, or three-body interactions) on lattices. We truncate the expansion in the first model using LASSO regularization, focusing on local interactions to prevent overfitting. In the second model, the expansion describes the CO adsorption energies and local CO-CO interactions obtained from machine learning (ML) regression models. We describe the model training and descriptors in detail in our previous work.[4] The overall Hamiltonian can be written as:

$$H(\boldsymbol{\sigma}) = H_{Pd_{n-i}/CeO_2}(\boldsymbol{\sigma_{Pd}}) + H^{CO-ads}_{Pd_{n-i}/CeO_2}(\boldsymbol{\sigma}_{CO}) \quad (1)$$

Here $\boldsymbol{\sigma}_{Pd}$ is the Pd_{n-i} cluster configuration, and $\boldsymbol{\sigma}_{CO}$ is the CO adlayer configuration. The overall configuration $\boldsymbol{\sigma}$ is the union of $\boldsymbol{\sigma}_{Pd}$ and $\boldsymbol{\sigma}_{CO}$.

Supplementary Note 3 - Lattice Representation of Structures

Each elementary event in the mechanism is represented by a graph on a lattice with an initial and final state; and each cluster pattern in the Hamiltonians is a subgraph of the lattice. In the case of bare Pd_n, the Pd lattice consists of 4 closest-packed layers above the support. Layer 0 (L0) is the base layer of Pd atoms on the support; layer 1 (L1) refers to Pd atoms just above it, and so on. Each unit cell (Fig. 1b) contains one L0, two L1, three L3, and three L4 sites. For Pd_n exposed to CO, we define a CO (the adsorbate) lattice consisting of top, bridge, and hollow sites, as indicated by DFT calculations for the CO adsorption locations. We give each CO adsorption site a unique label, consisting of the site type and the layer numbers of its neighboring Pd sites. For example, Hollow_L011 suggests that a CO adsorbs on a hollow site formed by a Pd atom in L0 and two Pd atoms in L1 layers. Each unit cell (Fig. 1c) contains one L0, two L1, and 28 CO adsorption sites in total (3 top, 12 bridge, and 13 hollow sites). Test simulations show that the formation of Pd clusters with L2 sites and higher are rarely occupied by CO. Therefore, we ignore the sites above layer 2 to reduce the simulation cost by half.

Supplementary Note 4 - Kinetic Monte Carlo Simulation Details

The forward and reverse rate constants are parameterized using an Arrhenius expression:

$$k_{fwd} = A_{fwd}\, exp(-\frac{E_{a,fwd}}{k_BT}) \quad (2)$$

$$k_{rev} = A_{rev}\, exp(-\frac{E_{a,fwd}-\Delta \mathrm{E}(\boldsymbol{\sigma})}{k_BT}) \quad (3)$$

Here k_B is the Boltzmann constant, and T is the temperature. $\Delta E(\boldsymbol{\sigma})$ is the energy difference between the initial and final state ($H_{init}(\boldsymbol{\sigma}) - H_{final}(\boldsymbol{\sigma}')$). A_{fwd} and A_{rev} are the forward and reverse pre-exponential factors. The activation energies ($E_{a,fwd}$) of diffusion events are computed *via* DFT. We assume CO adsorption is non-activated ($E_{a,fwd} = 0$) with an early 2D gas like transition state.[5,6] The pre-exponential factors of adsorption and desorption are calculated as:

$$A_{ads} = \frac{P_x A_{st}}{\sqrt{2\pi m_x k_B T}} \quad (4)$$

$$A_{des} = \frac{q_{vib,X(gas)} \cdot q_{rot,X(gas)} \cdot q_{trans-2D,X(gas)}}{q_{vib,X}} \frac{k_B T}{h} \quad (5)$$

Here P, A_{st}, m_x and h are the partial pressure of a species, the area of the adsorption site, the molecular mass and the Plank constant, respectively. q terms are the partition functions estimated from the vibrational frequencies and other thermodynamic properties.

Zacros implements a rejection-free algorithm with local updating of the propensities. The propensity is, in essence, the microscopic reaction rate of an event. The locality refers to a neighborhood of a site within a certain radius. Neighboring sites, including the absorbates, in this radius are the ones that affect the microscopic event rate on the specific site. In turn, these neighboring sites are affected by the event. The local update, also known as the list of neighbors, refers to updating only those sites and events on the picked site and its neighbors. This saves tremendous computational time in a KMC simulation. Rejection free refers to an algorithm where no null events happen, i.e., every Monte Carlo trial is successful. At each instant, possible events are generated, and one event is chosen with a probability proportional to its propensity.

The simulations require a lattice structure, an energetics model, and a reaction mechanism as input. For supported Pd subnanometer clusters, we consider: (1) bare Pd_n (CO pressure of 0 bar), and (2) Pd_n in a pressure of CO. We layout the energetics model and reaction mechanism for each case below. Supplementary Table S1 shows the symbols and notations for the sites and events used throughout this work.

Supplementary Table 1. Symbols and notations in the energetics and mechanisms.

Pd Lattice Sites			CO Adsorption Lattice Sites		
Site type	Occupied Sites	Vacant Sites Involved in Mechanism	Site type	Occupied Sites	Vacant Sites Involved in Mechanism
L0			Top		
L1			Bridge		
L2			Hollow		
L3			Gas Phase CO		
Sites not Involved					
Atomic Movement			Atoms in One Cluster		

Supplementary Note 5 - Case of Bare Pd_n

Energetics Model

The energy of (the ith isomer) the $Pd_{n\text{-}i}$ cluster of n Pd atoms on $CeO_2(111)$ support is parameterized by a cluster expansion (CE) Hamiltonian:

$$H_{Pd_{n-i}/CeO_2}(\boldsymbol{\sigma_{Pd}}) = N_{sites}J_e + \sum_x^{sites} J_x\sigma_x + \sum_{ij}^{pairs} J_{xy}\sigma_x\sigma_y + \sum_{xyz}^{triplets} J_{xyz}\sigma_x\sigma_y\sigma_z \quad (6)$$

The energy is given as a function of the state (or configuration), i.e., $\boldsymbol{\sigma_{Pd}}$, the occupancies over all Pd lattice sites, where $\sigma_x = 1$ (0), represents the presence (absence) of Pd atom at site x. N_{sites} is the total number of lattice sites and $N_{sites}J_e$ is the bias term. The effective cluster interaction (ECI) describes the energy contribution from each cluster pattern, including all lattice sites (J_e), the occupied sites (J_x), and interactions between pairs (J_{xy}), three-body (J_{xyz}) terms. The reference states are n single Pd atoms on $CeO_2(111)$ support. The ECIs are determined by fitting the CE to DFT energies. To prevent overfitting and speed up computations, we select 28 significant cluster patterns using LASSO regression as shown below.

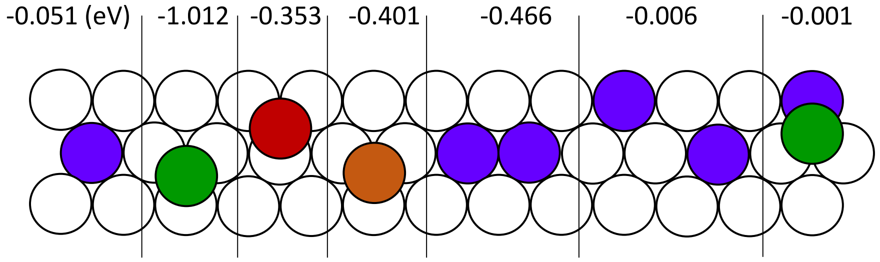

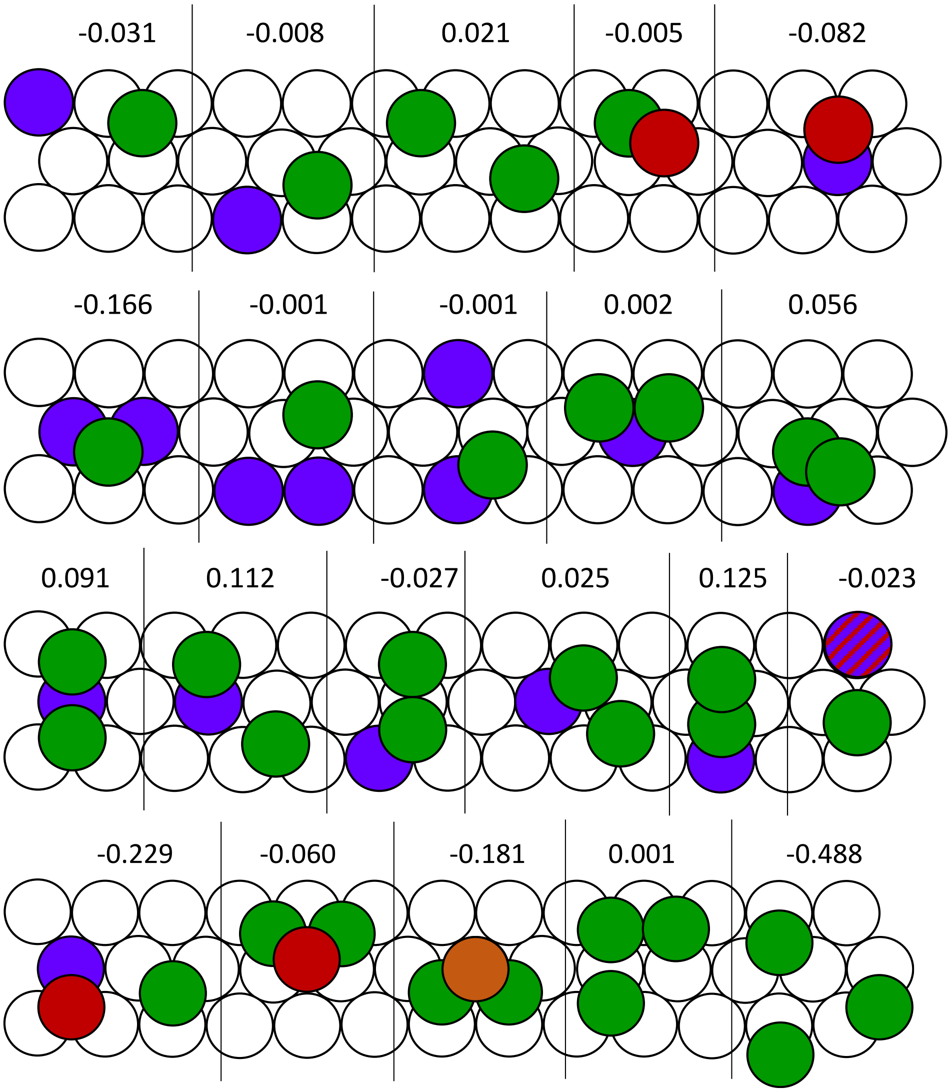

Supplementary Figure 11. 28 cluster patterns in the bare Pd_n system. The top row indicates corresponding ECI values in eV.

<u>Mechanism</u>

Starting from isolated single atoms, the agglomeration of Pd clusters can occur *via* diffusion events. From DFT calculations, we compute the diffusion barriers for Pd single atoms and small clusters on $CeO_2(111)$. The diffusion barrier increases with the cluster size: the barrier for Pd single atoms is 0.14 eV whereas the barrier for Pd_4 diffusion is 1.35 eV, making the latter only possible at higher temperatures. Thus, it is reasonable to eliminate larger cluster diffusion events from the mechanism as they are not kinetically relevant. We include diffusion events 1, 5, 6, 7, and 8 in Table S2 of single atoms and small cluster diffusion on the support. In addition, DFT calculations indicate that the Pd single atoms can easily diffuse on Pd atomic layers with a low barrier of 0.05 eV (events 2-4). This mechanism contributes to the rapid restructuring of local structure. Pd single atoms can also "hop" from a lower atomic layer to a higher one (events 9-11), contributing to vertical cluster growth. The activation barrier is determined to be 0.85 eV, much higher than the diffusion barrier in the same layer. In Supplementary Table 2 and Supplementary Fig. 12, all events are reversible, and their activation barriers are estimated from microscopic reversibility, i.e., the sum of ΔE (the energy difference between the initial and final state) and $E_{a,fwd}$. DFT calculations also

suggest that the attachment of free single atoms to other single atoms or clusters occurs fairly easily when they diffuse to nearby locations, i.e., their actual barrier is equal to the lower diffusion barrier of the participating species. Thus, we assume the barrier of attachment to be the diffusion barrier, and the barrier of detachment to be the sum of ΔE and the diffusion barrier, respectively. This is a common assumption made in diffusion-driven studies.[7] In this way, the detailed balance is maintained. An example of Pd_1 attachment to another Pd_1 forming a Pd_2 is illustrated in Supplementary Fig. 13. An entire cluster can diffuse on rare occasions and leave atoms off the support above the base layer. Thus, we include several barrierless events to drop the floating atoms back onto the support, ensuring the model is physically sound.

Supplementary Table 2. List of the elementary events included in the bare Pd_n system.

Index	Elementary event	Barrier E_a (e)	Prefactor A_{fwd} (s^{-1})	A_{ratio}
1	Pd_1 diffusion on layer 0	0.14	1.00E+13	1
2	Pd_1 diffusion on layer 1	0.05	1.00E+13	1
3	Pd_1 diffusion on layer 2	0.05	1.00E+13	1
4	Pd_1 diffusion on layer 3	0.05	1.00E+13	1
5	Pd_2 diffusion on layer 0	0.37	1.00E+13	1
6	Pd_3 diffusion on layer 0	0.71	1.00E+13	1
7	Pd_4 diffusion on layer 0	1.35	1.00E+13	1
8	Pd_4_3d diffusion on layer 0	1.35	1.00E+13	1
9	Pd_1 hopping from layer 0 to layer 1	0.85	1.00E+13	1
10	Pd_1 hopping from layer 1 to layer 2	0.85	1.00E+13	1
11	Pd_1 hopping from layer 2 to layer 3	0.85	1.00E+13	1

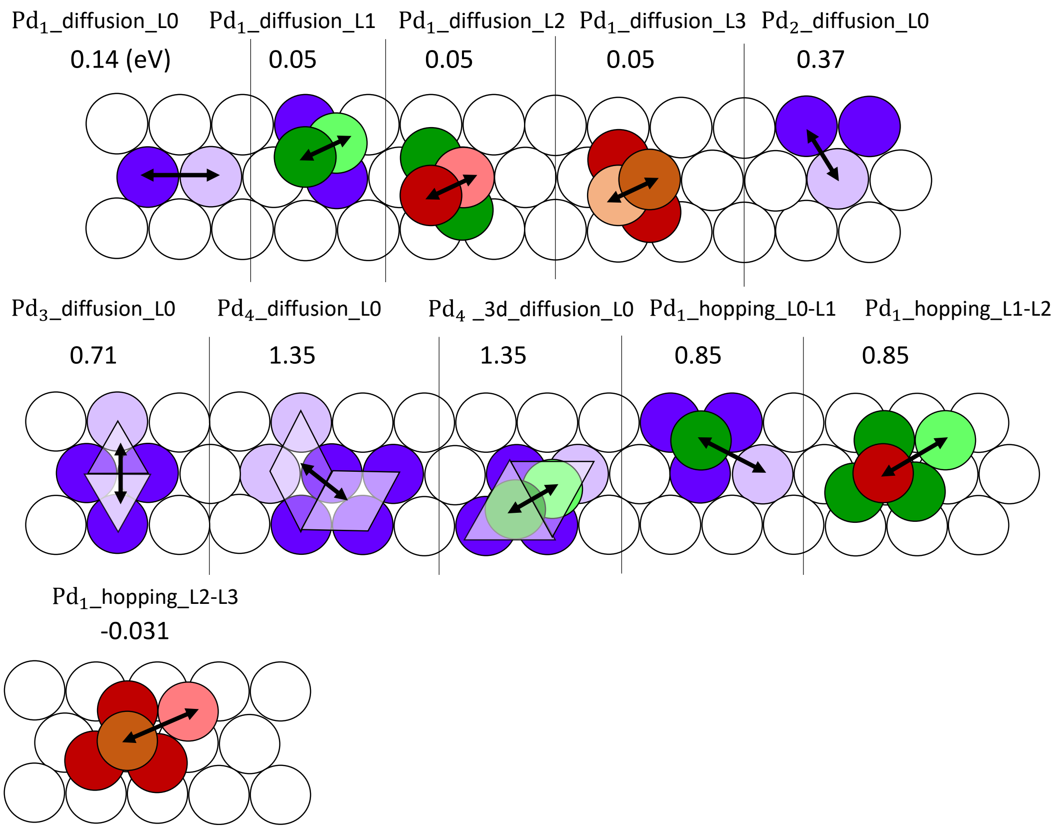

Supplementary Figure 12. Elementary events in the bare Pd_n system. The top row indicates corresponding forward barrier in eV.

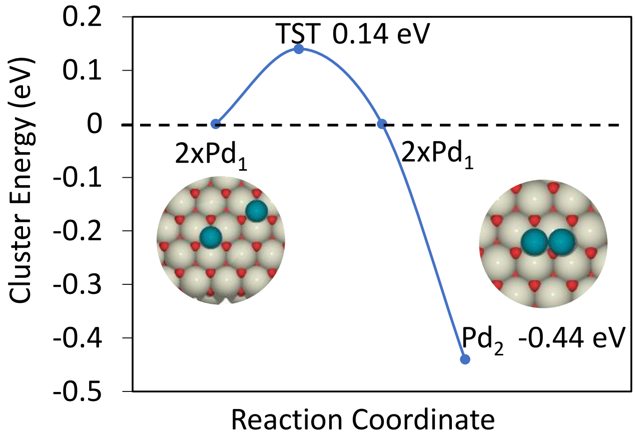

Supplementary Figure 13. Illustration of a single atom attachment (detachment) event. The forward event (Pd_2 formation) describes Pd_1 diffusion on layer 0 where Pd_1 attaches to another Pd_1. The reverse event (Pd_2 dissociation) describes Pd_1 detachment and diffusion. The forward and reverse barrier is 0.14 and 0.58 eV, respectively. The latter is computed from the initial state (2 Pd_1), the transition state (TST) and the final state (Pd_2) energy.

Supplementary Note 6 - Case of Pd_n exposed to CO

Next, we introduce the effect of CO adsorption into KMC simulations. We discuss CO-related cluster patterns and events in the energetics and mechanism below. It should be noted that only CO chemisorption on Pd atoms is considered. Our DFT calculations show that CO adsorption on the bare CeO_2(111) is weak with an adsorption energy of -0.20 eV. The distance between CO and the CeO_2(111) surface is 2.87 Å (Supplementary Fig. 14), indicating a typical physisorption. Therefore, we did not include it into the model.

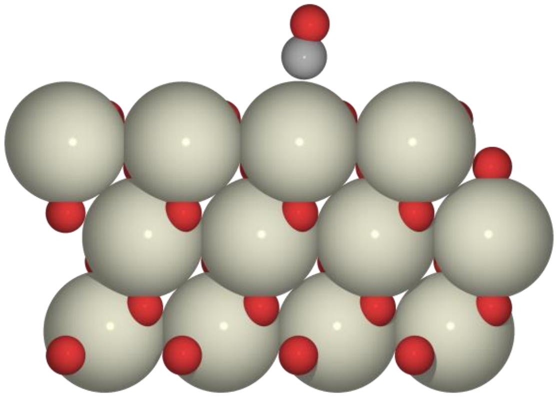

Supplementary Figure 14. CO physisorption on the bare CeO_2(111) surface.

Energetics Model

The overall Hamiltonian can be written as:

$$H(\boldsymbol{\sigma}) = H_{Pd_{n-i}/CeO_2}(\boldsymbol{\sigma_{Pd}}) + H^{CO-ads}_{Pd_{n-i}/CeO_2}(\boldsymbol{\sigma_{CO}}) \qquad (7)$$

Where the term ($H^{CO-ads}_{Pd_{n-i}/CeO_2}$) is the CO adlayer energy. It can be rewritten as a lattice-gas Hamiltonian that represents the energy of adsorption of m independent CO molecules (single molecule coverage limit) plus the lateral interaction between the molecules:

$$H^{mCO-ads}_{Pd_{n-i}/CeO_2}(\boldsymbol{\sigma_{CO}}) = \sum_x^{sites} J_x^{CO}\sigma_x + \sum_{xy}^{pairs} J_{xy}^{CO}\sigma_x\sigma_y \qquad (8)$$

Here $\boldsymbol{\sigma_{CO}}$ is the CO adlayer configuration in the CO lattice. The occupation variable $\sigma_x = 1$ (0) represents the presence (absence) of CO at site x. We consider the contributions of point- (single CO adsorption energy) and pair- (CO-CO lateral interactions) cluster patterns to the total energy. J_x^{CO}, J_{xy}^{CO} are the ECIs of a single CO and 2-body interactions with CO at site *y*, respectively.

The adsorption energy is primarily affected by the site-local environment. We use a descriptor-based machine learning (ML) model to correlate five geometric descriptors with the single CO adsorption energy in the previous work. The model is trained against DFT data. The adsorption energy at site *x* is expressed as:

$$E^{CO-ads^{(x)}}_{Pd_{n-i}/CeO_2} = f(n, S^{(x)}_{type}, CN1^{(x)}, CN2^{(x)}, Z^{(x)}) \qquad (9)$$

Where the descriptors are the cluster size n, the site type S_{type}, the first coordination number (CN1), the second coordination number (CN2), and the distance of each site from the support Z. In this work, we treat the ML model as ground truth and use it to generate single adsorption energies. To encode the ML model in Zacros while maintaining a low computational cost, we select a group of significant sites and then

translate them into graphs (i.e. cluster patterns) which Zacros could read. First, we include all the sites on smaller clusters ($n \leq 5$) in the energetics to run test simulations as they are dominant in the bare Pd_n simulations. After the test simulations are complete, we select the frequently observed sites on larger clusters as the new cluster patterns to add into the energetics. The smaller cluster patterns are usually subgraphs of larger cluster patterns. Therefore, the latter's ECI values are determined by subtracting the ML-model predicted value with the ECIs sum of the existing subgraphs. This process is performed iteratively until the error of Zacros model for larger clusters falls below a threshold. A threshold value of 0.15 eV for $n > 5$ (0.05 eV for $n \leq 5$) is chosen to balance computational cost and accuracy. Supplementary Fig. 15 shows 53 cluster patterns selected for single CO cluster patterns.

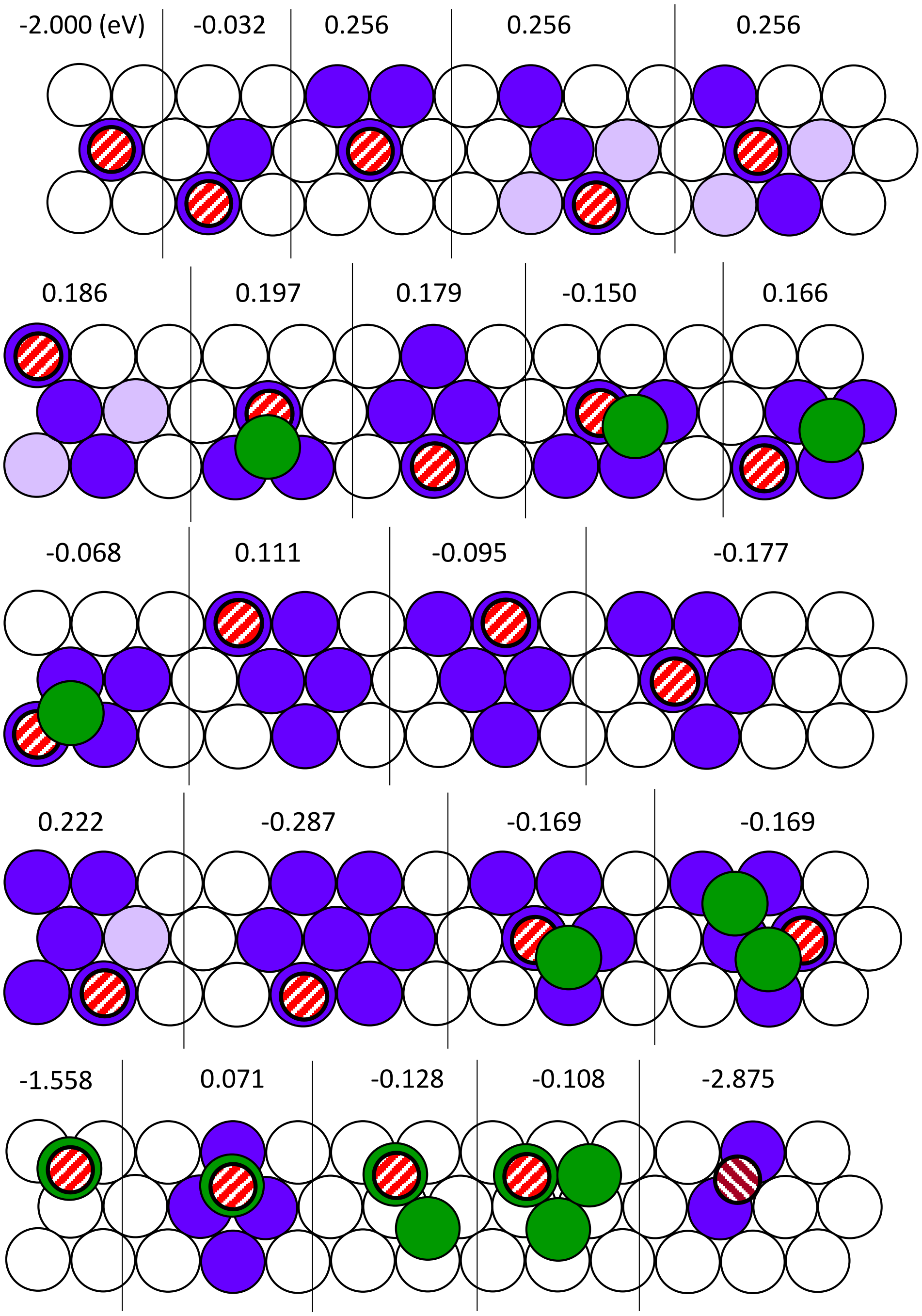

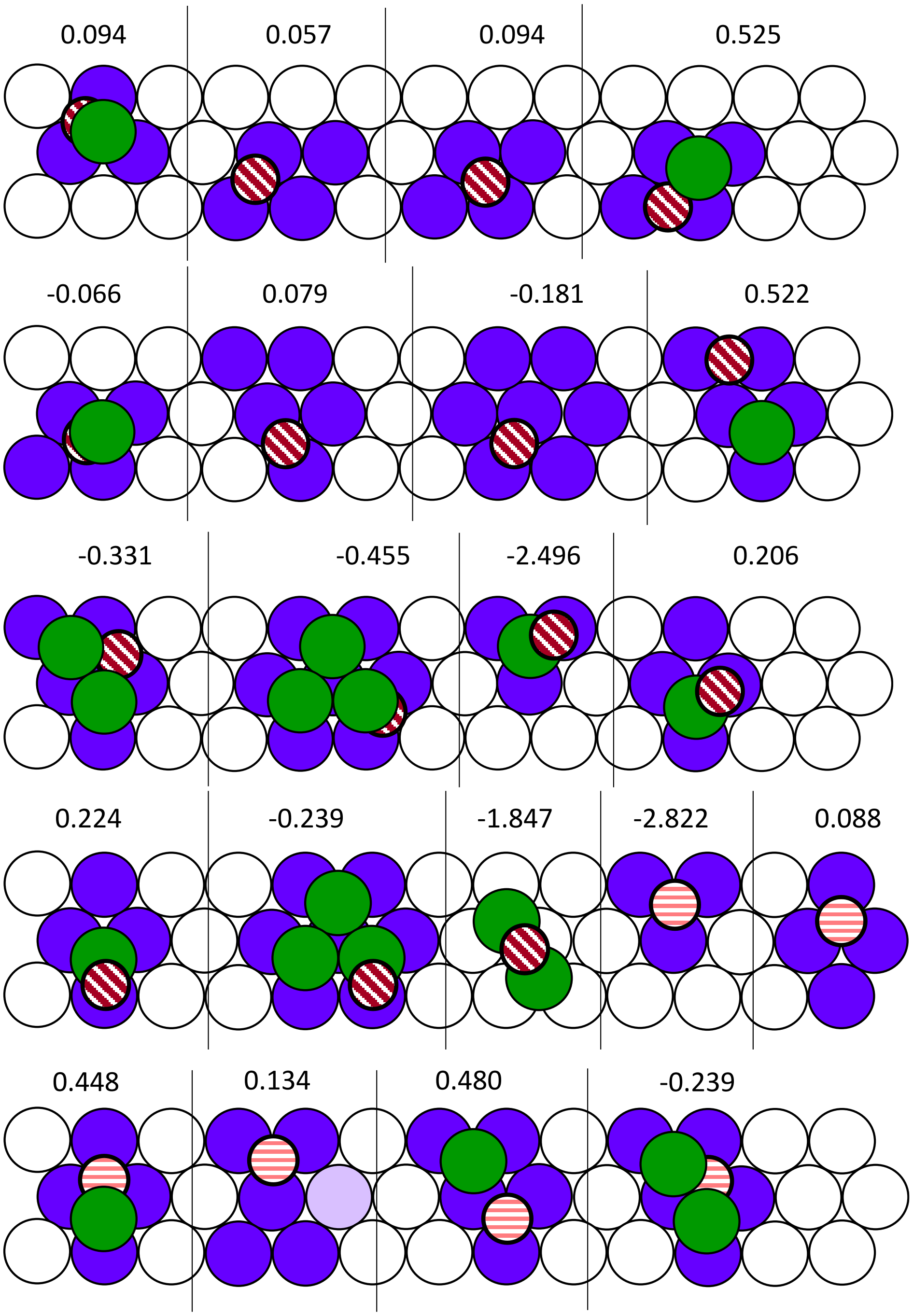
0.094
0.057
0.094
0.525
-0.066
0.079
-0.181
0.522
-0.331
-0.455
-2.496
0.206
0.224
-0.239
-1.847
-2.822
0.088
0.448
0.134
0.480
-0.239

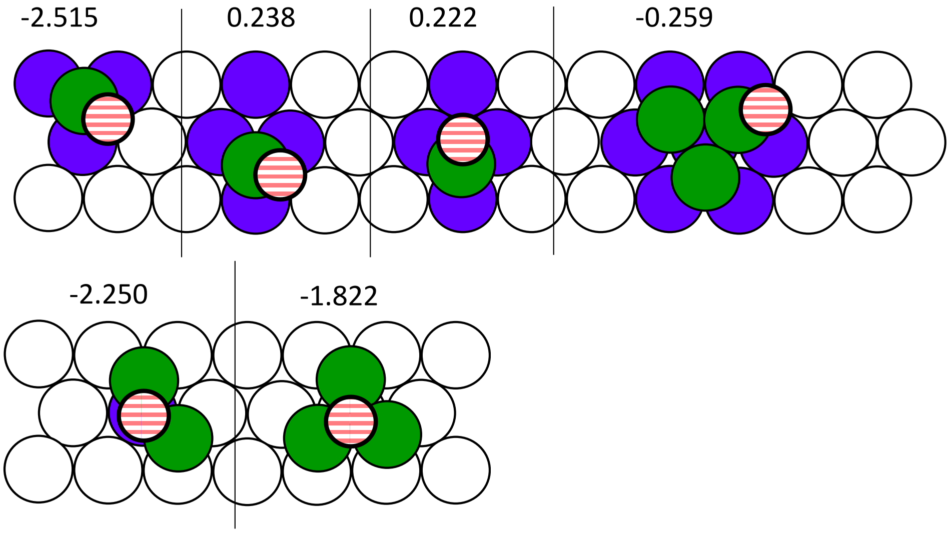

Supplementary Figure 15. 53 cluster patterns in the Pd-CO system for single CO adsorption. The top row indicates corresponding ECI values in eV.

Under experimental conditions, multiple CO adsorptions onto a cluster could occur and lead to high CO coverages. We add 27 2-body cluster patterns (Supplementary Fig. 16) into the energetics, representing the lateral interactions among CO-CO adsorbates on top, bridge, and hollow sites. The ECI values are determined from an ordinary least regression using the DFT dataset.

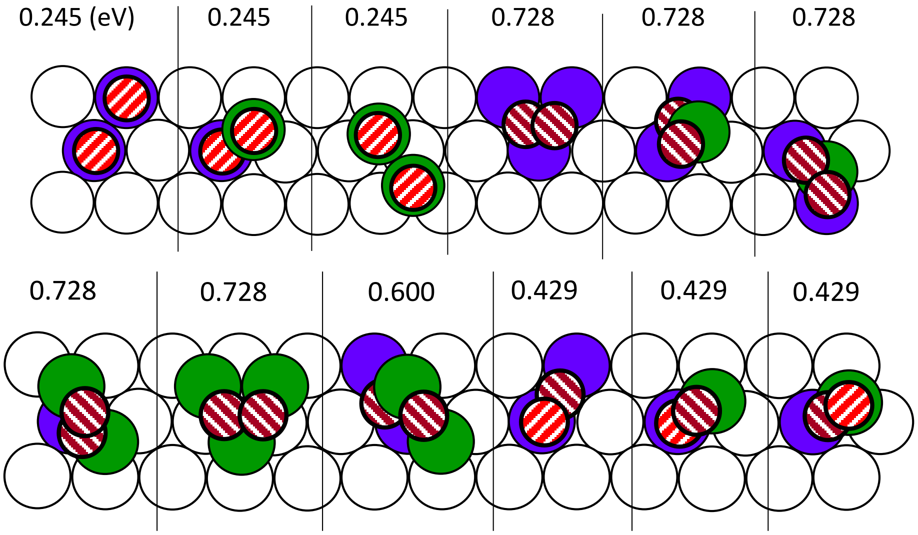

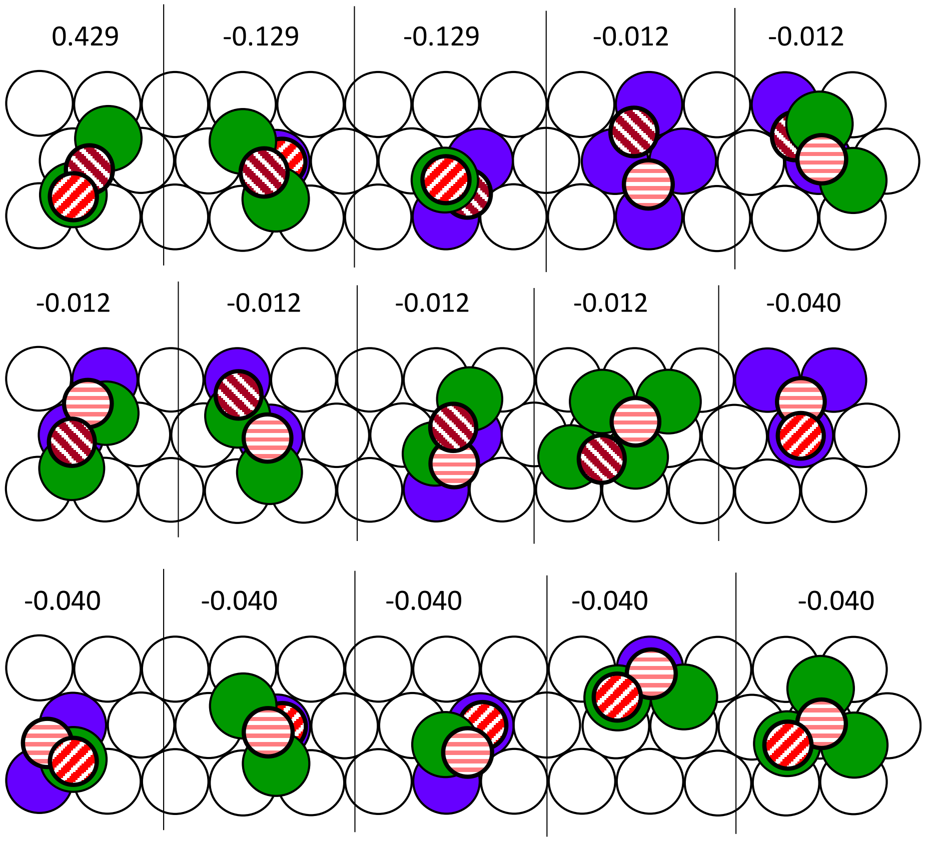

Supplementary Figure 16. 27 cluster patterns in the Pd-CO system for CO-CO interactions. The top row indicates corresponding ECI values in eV.

<u>Mechanism</u>

To introduce the effect of CO, we include adsorption events onto various sites of Pd and diffusion of CO-attached smaller clusters (n ≤ 4) into the model (Supplementary Table 3 and Supplementary Fig. 17). CO can absorb onto top, bridge, and hollow sites on each layer or between two layers (events 12-20). The events in Supplementary Fig. 5 are labeled based on the type of movement, the type of CO adsorption site, and the layer numbers of its neighboring Pd sites. DFT calculations also suggest that CO-attached clusters are mobile on the support, namely, the base layer L0. We compute the barrier for Pd_1(CO)-Pd_4(CO) diffusion (events 21-24) with CO placed on the most stable site on each cluster, i.e., Top_L0 on Pd_1, Bridge_L00 on Pd_2, Hollow_L000 on Pd_3, and Hollow_L001 on Pd_4-3d. The first three barrier values are significantly higher compared to the bare Pd_n diffusion of the same size. For instance, we see a 0.48 eV difference (0.14 eV vs. 0.62 eV) for Pd_1 diffusion vs. Pd_1(CO) diffusion, which indicates that CO adsorption stabilizes the free single atoms and makes them less mobile. For Pd_4-3d, a slight decrease in the barrier is observed upon adsorption. We include these events in the model.

Supplementary Table 3. List of additional elementary events included in the Pd-CO system.

Index	Elementary event	Barrier E_a (e)	Prefactor A_{fwd} (s^{-1})	A_{ratio}
12-20	CO adsorption on various sites	0	9.67E-02	5.20E-14
21	Pd_1(CO) diffusion on layer 0	0.62	1.00E+13	1
22	Pd_2(CO) diffusion on layer 0	0.52	1.00E+13	1
23	Pd_3(CO) diffusion on layer 0	1.17	1.00E+13	1

24	Pd_4_3d(CO) diffusion on yer 0	1.03	1.00E+13	1

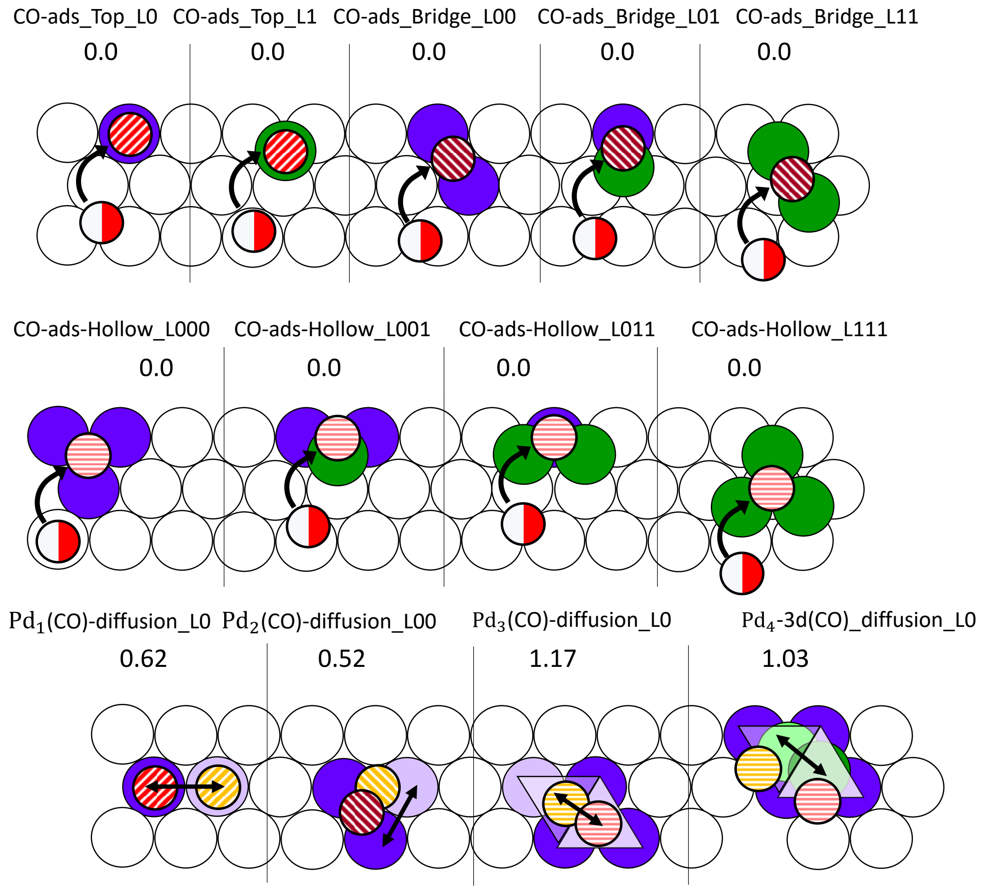

Supplementary Figure 17. Additional elementary events in the Pd-CO system. The top row indicates the corresponding forward barrier in eV.

Supplementary Note 7 - Acceleration of KMC

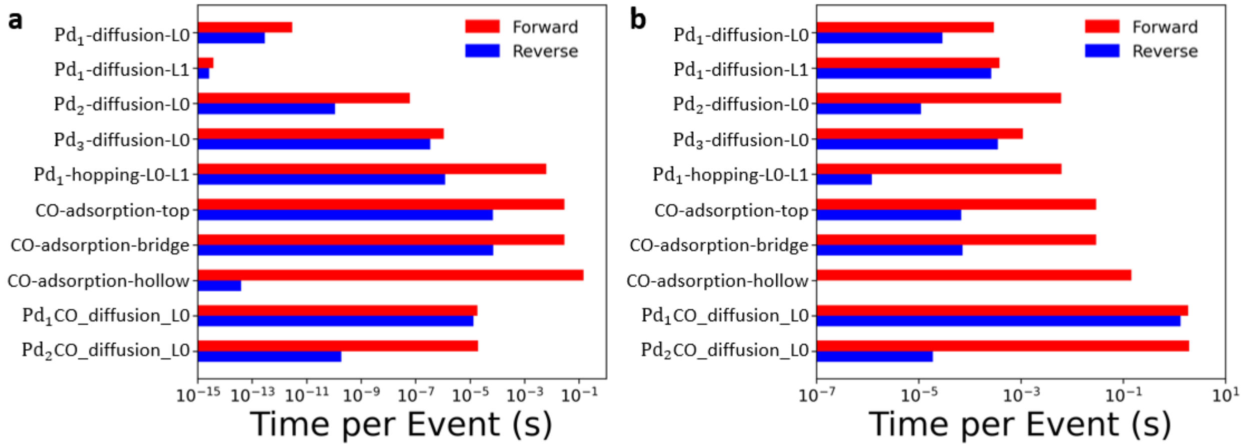

Supplementary Figure 18. Average time per event (a) before and (b) after pre-factor scaling in the simulation with an initial state of 20 single atoms at 300K, CO partial pressure of 0.1 bar at long times (over 168 hr).

The rate constants of diffusion of small clusters are much higher than those of other processes, namely, Pd atoms hopping between layers and CO adsorption. These fast events would quickly reach quasi-equilibrium and dominate the dynamics. The system would continue to simulate the frequent events without making advances in the time clock, leading to a waste of computational time. Acceleration can be achieved by scaling down rates of faster events. It has been demonstrated that scaling down the forward and reverse rate proportionally would incur a negligible error and preserve the thermodynamic properties.[8] In this work, we follow a similar approach laid out by Nunez *et al.*,[9] but the implementation is done in a supervised manner due to the complexity and high stiffness of the system. We first identify the faster events by running test simulations with several initial states with and without CO: isolated single atoms, small clusters, and CO-adsorbed small clusters. An example is shown in Supplementary Fig. 18a for the simulation with an initial state of 20 single atoms at 300 K. We adjust the forward pre-factors of Pd_1 and Pd_2 diffusion events explicitly (reduced by a factor up to 10^7) and leave the forward/reverse pre-factors ratio unchanged. After pre-factor scaling (Supplementary Fig. 18b), scaled events remain faster than other events.

As the relative time scales of events might evolve, we segment the simulations for statistical monitoring and control of stiffness. The goal is to ensure that slower events are adequately sampled while scaled events stay at least 100 times faster. Simulations are run over multiple segments, using the final states of previous trajectories as the initial states of new trajectories. In each iteration, the faster events are identified, and their forward pre-factors are reduced. In the Pd-CO case, Pd_1 hopping events are scaled too, whereas CO adsorption events are never scaled. We perform test simulations to ensure they produce clusters of similar sizes and shapes with and without scaling. An example is shown in Supplementary Fig. 19. For this simulation, each segment contains 14,000 KMC events, and the CPU time is around 40 hours on a single node of an Intel E5-2695 v4 processor. Initially, Pd_1 and Pd_2 diffusion on L0 dominates the dynamics, which leads to a small-time increment. In iteration 2, both events are scaled by a factor of 10^5. We see a time increment of 0.86 s. Pd_3 diffusion on L0 starts to occur while Pd_1 diffusion on L1 becomes the fastest event. In iteration 3, we scaled down Pd_1 diffusion on L1 by a factor of 10^5. The relative event frequencies remain unchanged, and the time increment is small, suggesting more aggressive scaling is needed. In iteration 4, Pd_1 diffusion on L1 is scaled by a factor of 10^4 again, and a few more Pd_1 hopping is observed. We see no significant structure changes of the Pd_4 cluster formed. This example indicates that pre-factor

scaling can effectively prioritize rare events and advance the time clock without altering the underlying dynamics.

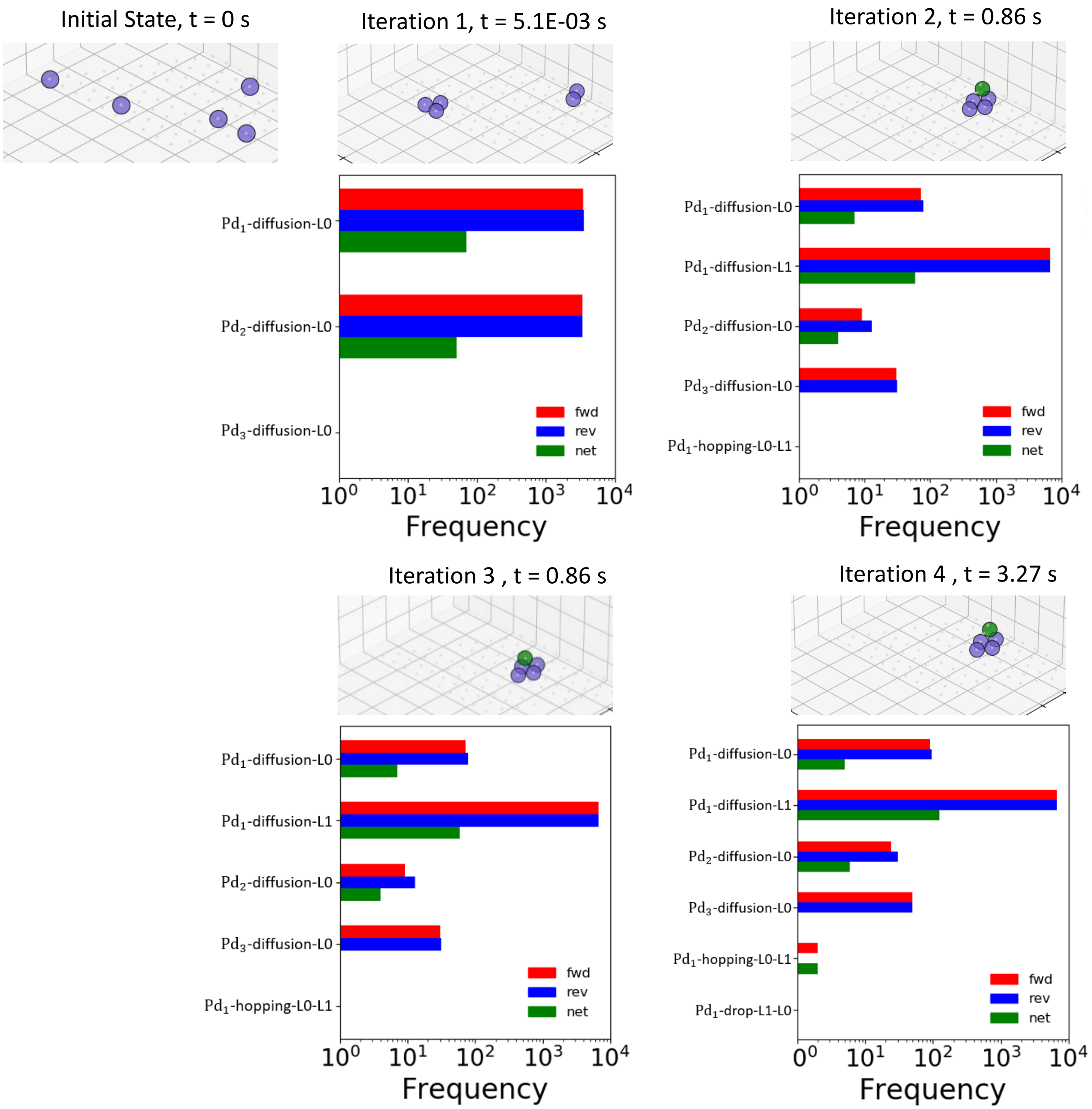

Supplementary Figure 19. Lattice snapshots and event frequency diagrams for each iteration in the simulation with an initial state of five single atoms at 300 K.

Supplementary Note 8 - Conversion between Metal Loadings and Lattice Coverages

The surface area of the support S and metal loading L (wt %) of the catalyst can be directly measured *via* experimental techniques during synthesis. Using the atomic mass m_{Pd} of the metal Pd and Avogadro number N_A, the surface atom concentration, namely, the number of Pd atoms per surface area, is,

$$C_{Pd} = \frac{N_A L}{m_{Pd} S} \quad (10)$$

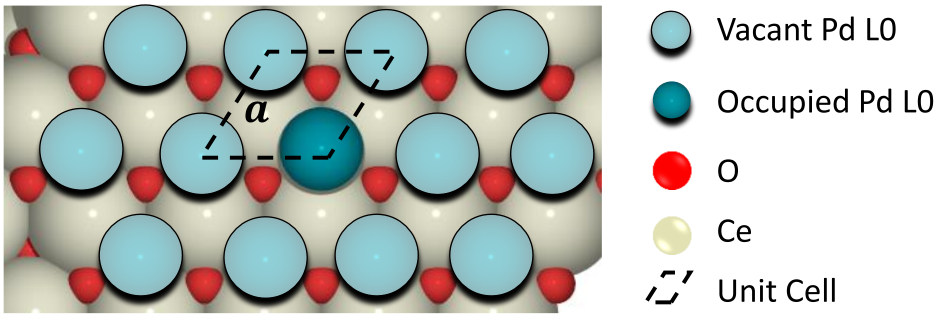

Supplementary Figure 20. Base layer (L0) of the bare Pd_n lattice.

Next, we assume three neighboring Pd atoms in L0 sites form an equilateral triangle (Supplementary Fig. 20). Given the Pd-Pd bond length a, the surface area of a unit cell in the lattice is:

$$S_{unit} = \frac{\sqrt{3}}{2} a^2 \quad (11)$$

The total number of Pd atoms in a unit cell is:

$$n_{Pd-unit} = C_{Pd} S_{unit} \quad (12)$$

Since there is only one Pd L0 site per unit cell, the coverage of Pd atoms on the base layer is also equal to $n_{Pd-unit}$. Combining equation (10)-(12), the coverage of Pd atoms can be written as a function of loading L:

$$Coverage = \frac{\sqrt{3} N_A a^2}{2 m_{Pd} S} L \quad (13)$$

Supplementary Table 4. Constants in the Pd_n/CeO2(111) system.

Notation	Constants	Values
N_A	Avogadro number	6.02×10^{23}
m_{Pd}	Atomic mass of Pd	106.42 g/mol
S	Surface area for a typical high-surface-area ceria	78 m^2/g
a	Pd-Pd bond length on CeO_2(111)	0.386 nm

Substituting the constant values (Supplementary Table 4) into equation (13), we obtain a relation converting metal loadings and lattice coverages:

$$Coverage = 0.09\, L \quad (14)$$

In addition, the coverage can be written as the ratio of the number of Pd atoms n and the total L0 sites (equal to the lattice size, n_{cell}):

$$Coverage = \frac{n}{n_{cell}} \quad (15)$$

The metal loading reported in the literature ranges from 0.1 wt% to 1 wt%.[10–12] Using equation (14), the coverage ranges from ~0.009 to 0.09. We use a 20 by 20 lattice size for most of the simulations. In the

single-atom case, using equation (15), the number of 8, 20, and 32 single atoms correspond to coverages of 0.02, 0.05, and 0.08. The loadings are 0.23, 0.57 wt%, 0.92 wt%, respectively, within reported experimental values. In the Pd_4_3d cluster case, the highest coverage tested is 0.1, and the loading is 1.15 wt%.

Supplementary References

1. Blöchl, P. E. Projector augmented-wave method. *Phys. Rev. B* **50**, 17953–17979 (1994).
2. Perdew, J. P., Burke, K. & Ernzerhof, M. Generalized Gradient Approximation Made Simple. *Phys. Rev. Lett.* **78**, 1396–1396 (1997).
3. Su, Y., Liu, J., Filot, I. A. W. & Hensen, E. J. M. Theoretical Study of Ripening Mechanisms of Pd Clusters on Ceria. *Chem. Mater.* **29**, 9456–9462 (2017).
4. Wang, Y., Su, Y.-Q., Hensen, E. J. M. & Vlachos, D. G. Finite-Temperature Structures of Supported Subnanometer Catalysts Inferred via Statistical Learning and Genetic Algorithm-Based Optimization. *ACS Nano* **14**, 13995–14007 (2020).
5. Pineda, M. & Stamatakis, M. Beyond mean-field approximations for accurate and computationally efficient models of on-lattice chemical kinetics. *J. Chem. Phys.* **147**, 024105 (2017).
6. Reuter, K. First-Principles Kinetic Monte Carlo Simulations for Heterogeneous Catalysis: Concepts, Status, and Frontiers. in *Modeling and Simulation of Heterogeneous Catalytic Reactions* (ed. Deutschmann, O.) 71–111 (Wiley-VCH Verlag GmbH & Co. KGaA, 2011). doi:10.1002/9783527639878.ch3
7. Xu, L., Campbell, C. T., Jónsson, H. & Henkelman, G. Kinetic Monte Carlo simulations of Pd deposition and island growth on MgO(1 0 0). *Surf. Sci.* **601**, 3133–3142 (2007).
8. Stamatakis, M. & Vlachos, D. G. Equivalence of on-lattice stochastic chemical kinetics with the well-mixed chemical master equation in the limit of fast diffusion. *Comput. Chem. Eng.* **35**, 2602–2610 (2011).
9. Núñez, M., Robie, T. & Vlachos, D. G. Acceleration and sensitivity analysis of lattice kinetic Monte Carlo simulations using parallel processing and rate constant rescaling. *J. Chem. Phys.* **147**, 164103 (2017).
10. Jones, J. *et al.* Thermally stable single-atom platinum-on-ceria catalysts via atom trapping. *Science* **353**, 150–154 (2016).
11. Kunwar, D. *et al.* Stabilizing High Metal Loadings of Thermally Stable Platinum Single Atoms on an Industrial Catalyst Support. *ACS Catal.* **9**, 3978–3990 (2019).
12. Spezzati, G. *et al.* CO oxidation by Pd supported on CeO2(100) and CeO2(111) facets. *Appl. Catal. B Environ.* **243**, 36–46 (2019).
