# Supplementary material for: Real-time dynamics and structures of supported subnanometer catalysts via multiscale simulations
Source: Nat Commun. 2021 Sep 14;12:5430. doi: 10.1038/s41467-021-25752-8 (PMC8440615; doi:10.1038/s41467-021-25752-8)
Supplement: Supplementary file 3 — Description of Additional Supplementary Files [file 41467_2021_25752_MOESM3_ESM.pdf]

Description of Additional Supplementary Files

File Name: Supplementary Movie 1
Description: Lattice animation for 125 Pd single atoms on a 50 by 50 lattice (coverage = 0.05) in a vacuum.

File Name: Supplementary Movie 2
Description: Lattice animation for 125 Pd single atoms on a 50 by 50 lattice (coverage = 0.05) in a CO pressure of 0.1 bar.

File Name: Supplementary Movie 3
Description: Lattice animation for 20 Pd single atoms on a 20 by 20 lattice (coverage = 0.05) in a vacuum.

File Name: Supplementary Movie 4
Description: Lattice animation for 20 Pd single atoms on a 20 by 20 lattice (coverage = 0.05) in a vacuum.

File Name: Supplementary Movie 5
Description: Lattice animation for 6 Pd4_3d clusters on a 20 by 20 lattice (coverage = 0.06) in a vacuum.

File Name: Supplementary Movie 6
Description: Lattice animation for 6 Pd4_3d clusters on a 20 by 20 lattice (coverage = 0.06) in a CO pressure if 0.1 bar.

File Name: Supplementary Movie 7
Description: Lattice animation for a Pd_{20} cluster in a vacuum.
